# Supplementary material for: Atomistic Mechanisms of Stress-Dependent Molten Salt Corrosion in NiCr Alloys
Source: ACS Omega. 2026 Jun 16;11(25):37884–91. doi: 10.1021/acsomega.6c03055 (PMC13325345; doi:10.1021/acsomega.6c03055)
Supplement: Supplementary file 1 [file ao6c03055_si_001.pdf]

Supporting Information

Atomistic Mechanisms of Stress-Dependent Molten Salt Corrosion in NiCr Alloys

Hamdy Arkoub[†], Jia-Hong Ke[‡], Miaomiao Jin[†,*]

†*Department of Nuclear Engineering, The Pennsylvania State University, University Park, PA 16802, USA*

‡*Computational Mechanics and Materials Department, Idaho National Laboratory, Idaho Falls, ID, 83415, USA*

E-mail: mmjin@psu.edu

S1. Grain Boundary and surface Energy Calculations

Grain boundary and surface energy calculations were performed for pure Ni and $Ni_{0.75}Cr_{0.25}$. Three atomistic models were constructed: a bicrystal grain boundary (GB) model, a bulk reference model, and a free surface (FS) slab model, as shown in Figure S1. The GB model consists of a periodic bicrystal containing two equivalent Σ5(210)[001] grain boundaries. The FS model was constructed using the same crystallographic orientation, with 20 Å vacuum introduced along [210] direction. All three models contain 640 atoms, and their dimensions are listed in Table S1. All structures were optimized using the Amsterdam Modeling Suite (AMS) with the same ReaxFF potential used in the corrosion simulations [1-4]. Geometry optimization was performed using convergence thresholds of 10^{-6} Hartree for the energy change, 10^{-4} Hartree/Å for the maximum Cartesian gradient, and 0.001 Å for the maximum Cartesian step.

The grain boundary energy was calculated as:

$$\gamma_{\mathrm{GB}} = \frac{E_{\mathrm{GB}} - NE_{\mathrm{bulk}}}{2A} \qquad (1)$$

where E_{GB} is the total energy of the bicrystal containing two equivalent grain boundaries, N is the number of atoms, E_{bulk} is the bulk energy per atom, and A is the grain boundary area. The free surface energy was calculated as:

$$\gamma_{\mathrm{surf}} = \frac{E_{\mathrm{slab}} - NE_{\mathrm{bulk}}}{2A} \qquad (2)$$

Where E_{slab} is the total energy of the slab containing two free surfaces and A is the surface area. The factor of 2 accounts for the two exposed surfaces. For consistency with the Σ5(210) GB plane, the Ni(210) surface was used for the surface energy calculation, as shown in Figure S1c.

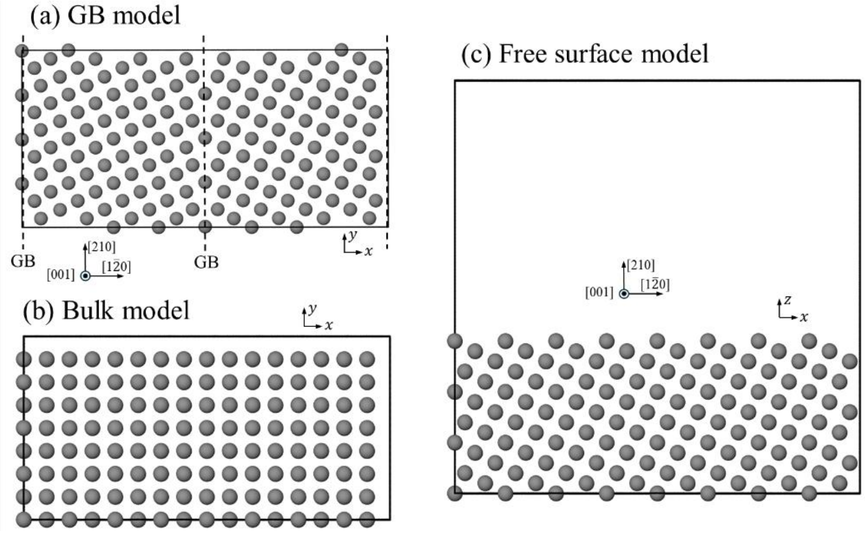

Figure S1: (a) Σ5(210)[001] GB bicrystal model containing two equivalent grain boundaries, (b) bulk reference model, and (c) FS slab model with two equivalent Ni(210) free surfaces.

Table S1: Dimensions of the atomistic models used for GB and surface energy calculations.

**Model**	**Dimensions (Å^3)**
GB model	32.29 × 15.62 × 13.91
FS model	31.48 × 17.60 × 31.81
Bulk model	28.16 × 17.60 × 14.08

References

[1] Van Duin, A. C., Dasgupta, S., Lorant, F., & Goddard, W. A. (2001). ReaxFF: a reactive force field for hydrocarbons. *The Journal of Physical Chemistry A*, *105*(41), 9396-9409.

[2] Chenoweth, K., Van Duin, A. C., & Goddard, W. A. (2008). ReaxFF reactive force field for molecular dynamics simulations of hydrocarbon oxidation. *The Journal of Physical Chemistry A*, *112*(5), 1040-1053.

[3] Van Duin, A. C. T., Goddard, W. A., Islam, M. M., van Schoot, H., Trnka, T., Yakovlev, A. L., & SCM. (2026). *ReaxFF* (Version 2026.1) [Computer software]. Theoretical Chemistry, Vrije Universiteit Amsterdam. https://www.scm.com

[4] Arkoub, H., Dwivedi, S., van Duin, A. C., & Jin, M. (2024). A reactive force field approach to modeling corrosion of NiCr alloys in molten FLiNaK salts. Applied Surface Science, 655, 159627.

S2. Stress Loading and Strained Model Dimensions

To generate strained alloy models, we stretched and compressed the NiCr slab along x direction at 800 °C. A constant engineering strain rate of 10^{-5} ps^{-1} was imposed under the NVT ensemble, with periodic boundary conditions in x and y and non-periodic boundaries in z. As shown in Figure S2, the resulting engineering stress–strain response remains linear up to ±4% strain, confirming that all selected configurations remain within the elastic regime.

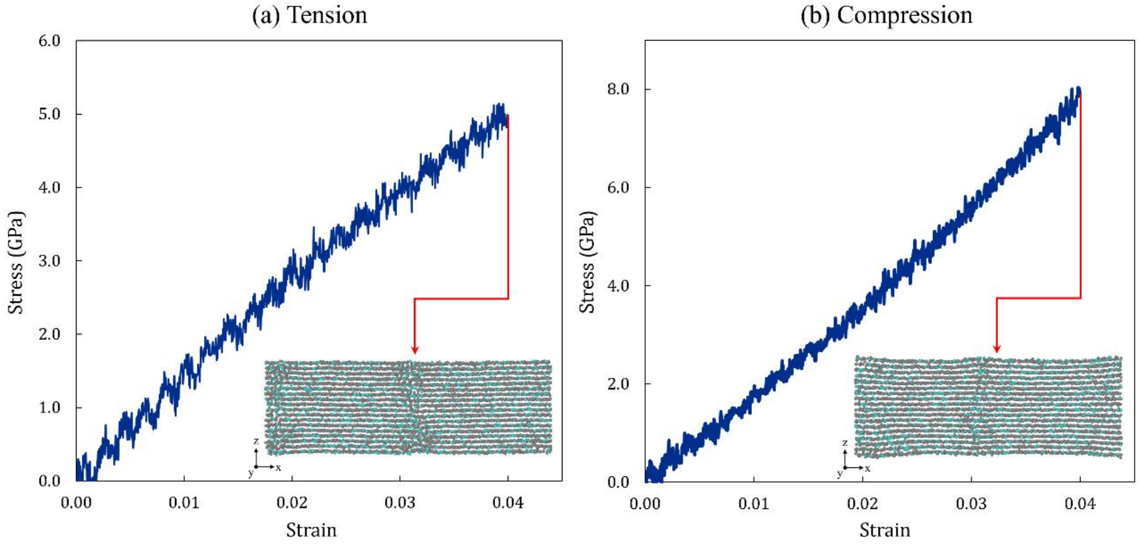

Figure S2: Engineering stress–strain response of the $Ni_{0.75}Cr_{0.25}$ slab under (a) tensile and (b) compressive loading along the x-direction at 800 °C. Representative atomic configurations at the final strain state are shown in the insets.

After introducing the equilibrated molten salt above the alloy surface, the final corrosion simulation cell dimensions for the tensile, compressive, and unstrained configurations are summarized in Table S2.

To verify that the observed stress-dependent corrosion behavior is not limited to the ±4% strain cases, additional corrosion simulations were performed using lower uniaxial strain states of ±1%. As shown in Figure S3, the same qualitative behavior is observed after 500 ps of corrosion. Under +1% tensile strain, localized penetration along the Σ5 grain boundary remains visible, but the extent of GB recession is reduced compared with the +4% case. Under −1% compressive strain, a localized ridge-like protrusion also develops near the GB, although it is smaller than that observed under −4% strain. The ±4% strain cases therefore provide amplified but mechanistically consistent stress-corrosion behavior within the short RMD simulation timescale.

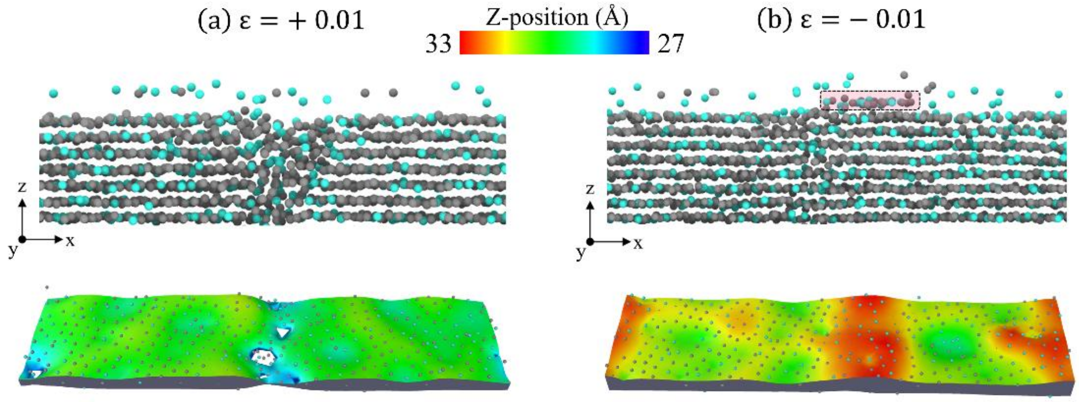

Figure S3: Zoomed-in atomic snapshots (top) and surface meshes (bottom) of the $Ni_{0.75}Cr_{0.25}$ Σ5(210) GB slabs after 500 ps of exposure to molten FLiNaK under (a) tensile strain value of +0.01 and (c) compressive strain value of –0.01. Surface meshes are colored by z-position, while Ni and Cr atoms are shown in gray and cyan, respectively.

Table S2: Simulation cell dimensions for the three elastic strain states used in the corrosion simulations: tensile, compressive, and unstrained.

Strain Models	Size (Å)
Unstrained (0.00)	92.45 × 24.07 × 48.36
Tensile Strain (+0.04)	100.15 × 24.07 × 47.05
Compressive Strain (-0.04)	96.30 × 24.07 × 49.33
Tensile Strain (+0.01)	97.26 × 24.07 × 48.11
Compressive Strain (-0.01)	95.34 × 24.07 × 48.64

S3. Evolution of Metal Atom Dissolution

Figure S4 shows the time evolution of the number of dissolved metal atoms per unit surface area during corrosion at 800 °C. Over the 500 ps simulation window, the total dissolved atom density remains comparable across the three conditions. In contrast, dissolution originating from the GB region is consistently about twice that of the bulk region, demonstrating clear preferential intergranular attack.

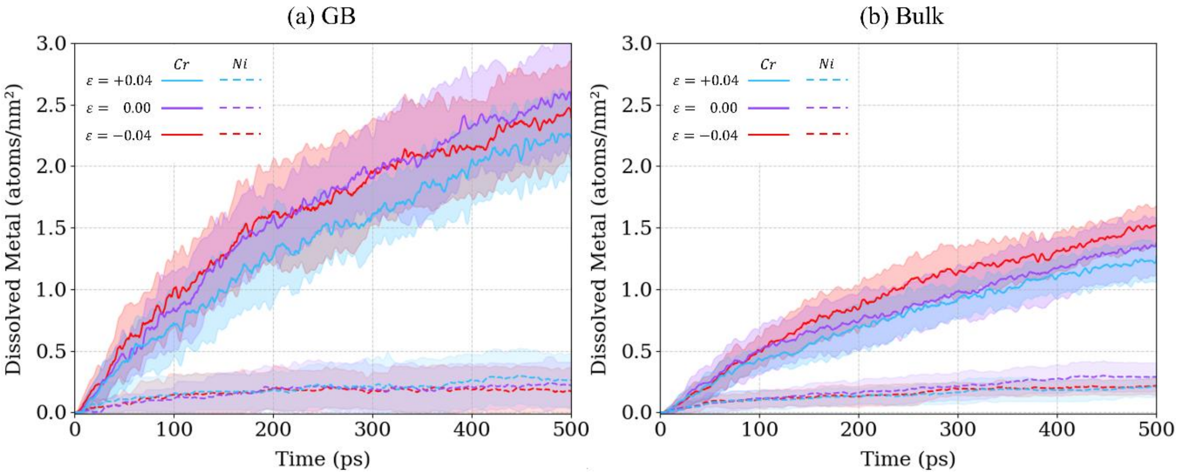

Figure S4: Time evolution of dissolved metal atoms per unit surface area during corrosion at 800 °C for (a) GB and (b) bulk regions under tensile, unstrained, and compressive conditions.

S4. Atomic Charge Distribution After Corrosion

Figure S5 presents the Ni charge distributions in the bulk and GB regions under the three strain conditions after 500 ps of corrosion. Ni atoms exhibit behavior similar to that observed for Cr in Figure 4 of the main manuscript, with charge accumulation localized near the salt–metal interface and more pronounced redistribution in the GB region compared to the bulk.

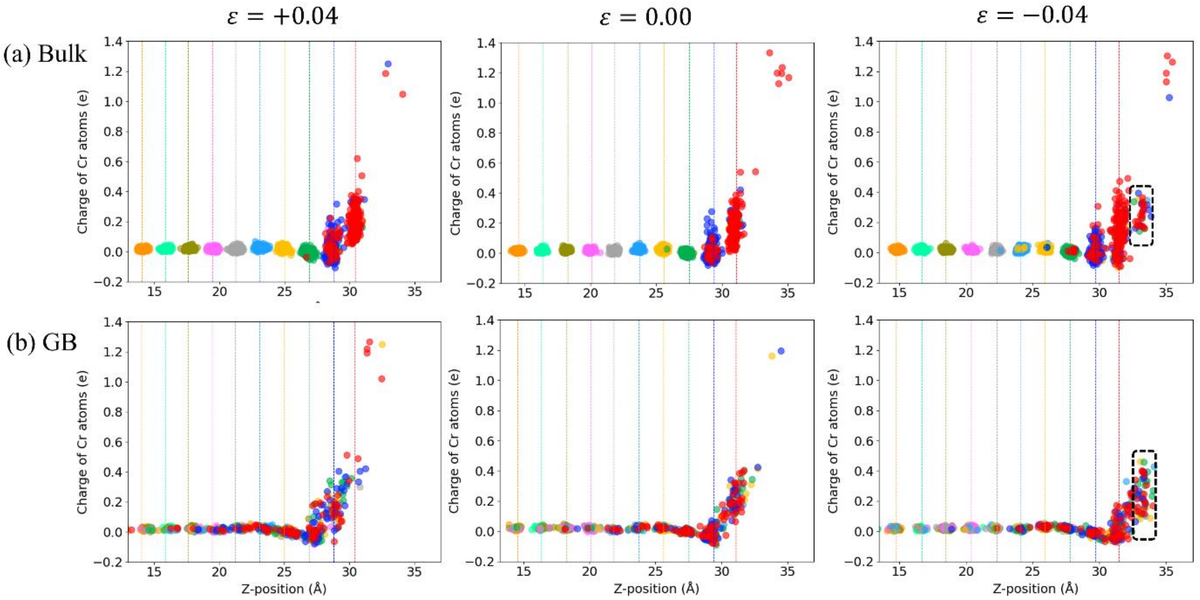

Figure S5: Distribution of Ni atomic charges along the z-direction after 500 ps of corrosion for the three strain states. (a) Top panels show bulk regions, while (b) bottom panels correspond to GB regions. Points are colored according to each atom's original layer in the pristine structure, and vertical dashed lines mark the initial layer positions.

S5. Grain Boundary Diffusivity Calculations

To quantify how strain-induced structural variations affect atomic mobility, the mean square displacement (MSD) of GB region Ni and Cr atoms was calculated with diffusion coefficients extracted from the linear MSD time regime using Eqs. (3)–(4).

$$MSD = \frac{1}{N}\left\langle \sum_{i=1}^{N} \left(r_i(t) - r_i(0)\right)^2 \right\rangle \qquad (3)$$

$$D = \frac{1}{2d}\frac{d}{dt}\left\langle \left(r_i(t) - r_i(0)\right)^2 \right\rangle \qquad (4)$$

Here, N is the number of atoms included in the MSD calculation, d is the dimensionality ($d = 3$), and $r_i(t)$ is the position of atom i at time t. The extracted diffusion coefficients are shown in Table S2.

Table S3: GB diffusion coefficients of Ni and Cr atoms under salt-exposed and salt-free conditions for the three strain states.

Applied Strain	With FLiNaK D (cm^2/s)		Salt Free D (cm^2/s)	
	Ni	Cr	Ni	Cr
Tensile Strain (+0.04)	8.42×10^{-7}	6.85×10^{-7}	4.89×10^{-7}	4.74×10^{-7}
Compressive Strain (-0.04)	6.63×10^{-7}	6.32×10^{-7}	4.05×10^{-7}	3.85×10^{-7}
Unstrained (0.00)	6.56×10^{-7}	5.51×10^{-7}	3.78×10^{-7}	3.49×10^{-7}
